# GCN2-Like Kinase Modulates Stress Granule Formation During Nutritional Stress in *Trypanosoma cruzi*

**DOI:** 10.3389/fcimb.2020.00149

**Published:** 2020-04-16

**Authors:** Amaranta Muniz Malvezzi, Mirella Aricó, Normanda Souza-Melo, Gregory Pedroso dos Santos, Paula Bittencourt-Cunha, Fabiola Barbieri Holetz, Sergio Schenkman

**Affiliations:** ^1^Departamento de Microbiologia, Imunologia e Parasitologia, Escola Paulista de Medicina, Universidade Federal de São Paulo, São Paulo, Brazil; ^2^Instituto Carlos Chagas, Fundação Oswaldo Cruz-Fiocruz, Curitiba, Brazil

**Keywords:** GCN2, eIF2α, *Trypanosoma cruzi*, TcK1, phosphorylation, stress-granules, CRISPR/Cas9

## Abstract

The integrated stress response in eukaryotic cells is an orchestrated pathway that leads to eukaryotic Initiation Factor 2 alpha subunit (eIF2α) phosphorylation at ser51 and ultimately activates pathways to mitigate cellular damages. Three putative kinases (*Tc*k1, *Tc*k2, and *Tc*k3) are found in the *Trypanosoma cruzi* genome, the flagellated parasite that causes Chagas disease. These kinases present similarities to other eukaryotic eIF2α kinases, exhibiting a typical insertion loop in the kinase domain of the protein. We found that this insertion loop is conserved among kinase 1 of several *T. cruzi* strains but differs among various Kinetoplastidae species, suggesting unique roles. Kinase 1 is orthologous of GCN2 of several eukaryotes, which have been implicated in the eIF2α ser51 phosphorylation in situations that mainly affects the nutrients levels. Therefore, we further investigated the responses to nutritional stress of *T. cruzi* devoid of *Tc*K1 generated by CRISPR/Cas9 gene replacement. In nutrient-rich conditions, replicative *T. cruzi* epimastigotes depleted of *Tc*K1 proliferate as wild type cells but showed increased levels of polysomes relative to monosomes. Upon nutritional deprivation, the polysomes decreased more than in *Tc*K1 depleted line. However, eIF2α is still phosphorylated in *Tc*K1 depleted line, as in wild type parasites. eIF2α phosphorylation increased at longer incubations times, but KO parasites showed less accumulation of ribonucleoprotein granules containing ATP-dependent RNA helicase involved in mRNA turnover (DHH1) and Poly-A binding protein (PABP1). Additionally, the formation of metacyclic-trypomastigotes is increased in the absence of *Tc*k1 compared to controls. These metacyclics, as well as tissue culture trypomastigotes derived from the *Tc*K1 knockout line, were less infective to mammalian host cells, although replicated faster inside mammalian cells. These results indicate that GCN2-like kinase in *T. cruzi* affects stress granule formation, independently of eIF2α phosphorylation upon nutrient deprivation. It also modulates the fate of the parasites during differentiation, invasion, and intracellular proliferation.

## Introduction

The formation of the ternary complex is the first step of the cap-dependent translation initiation process and comprises the eukaryotic initiation factor 2 (eIF2), the methionine-tRNA^i^ and a GTP molecule. eIF2 is a heterotrimeric complex that includes the eIF2α subunit, which can be phosphorylated at Ser 51; eIF2β, which binds to tRNA^met^, mRNA and others initiation factors; and eIF2γ that binds to the GTP and possesses GTPase activity (Hinnebusch and Lorsch, 2012). This complex scans the mRNA to find the AUG initiation codon and delivers the methionine for protein synthesis initiation (Jennings and Pavitt, 2014). For this, the ternary complex associates with the 40S minor ribosomal subunit and other translation factors such as eIF1, eIF1A, eIF3, and eIF5 to form the 43S pre-initiation complex. This complex binds to the mRNA cap region pre-activated by the eIF4F trimer, with the participation of the poly-A binding protein, eIF4B, eIF4H, and eIF3. Once bound near the cap, the 43S complex initiates mRNA scanning from the 5′-3′ direction until the recognition of the AUG initiation codon, which causes the hydrolysis of eIF2-linked GTP through its GTPase activity by eIF5, a GTPase-activating protein. eIF2-GDP is then released by the action of eIF5B ensuing translation elongation. In order to begin a new phase of translation initiation, the eIF2B protein converts GDP to GTP through its guanine nucleotide exchange factor activity (Gordiyenko et al., 2014).

Translation initiation is regulated through decreasing the levels of eIF2-GTP and by eIF4F complex availability. For the eIF2, phosphorylation of the α subunit at serine 51 results in an increased affinity of eIF2–eIF2B, preventing the GDP-GTP exchange (Jennings and Pavitt, 2014). This results in protein translation inhibition due to the unavailability of active ternary complex (Sudhakar et al., 2000). Four protein kinases were described as acting on eIF2α phosphorylation in eukaryotes. The protein kinase named General Control Non-repressible 2 (GCN2), which is activated in the presence of uncharged tRNAs and nutritional stress (Dever and Hinnebusch, 2005); the Heme Regulated Inhibitor (HRI) kinase, which acts in response to low level of intracellular heme (Chen and London, 1995); the RNA-dependent protein kinase (PKR), which is activated in response to viral infections (Garcia et al., 2007); and PKR-like endoplasmic reticulum kinase (PERK), activated in response to elevated levels of misfolded proteins (Ron and Harding, 2012). Since a large variety of stress stimuli ultimately lead to eIF2α phosphorylation, this pathway was denominated as the Integrated Stress Response. This pathway is crucial for cell survival, acting like a hub leading to eIF2α phosphorylation, reducing translation globally, and activating the translation of a particular subset of mRNAs that can revert the initial harm and restore regular levels of translation (Holcik and Sonenberg, 2005). In this scenario, selective mRNA translation occurs preferentially when small reading phases near the initiation codon are present and these proteins act modulating the stress response (Palam et al., 2011).

Trypanosomatids, a group of several protozoan parasites display unique gene expression control, characterized mainly by post-transcriptional regulation (Clayton, 2019). It is particularly relevant the control of mRNA stability due to the presence of several RNA binding proteins (Kolev et al., 2014; Harvey et al., 2018) and the formation of RNA granules (Cassola, 2011; Guzikowski et al., 2019), operating to cope with the environmental changes during the parasite life cycle. It is less known whether the regulation of translation initiation through eIF2α is also relevant for parasite adaptation. In Trypanosomatids, eIF2α has an N-terminal extension so the conserved Ser 51 corresponds to the amino acid threonine 169, as described in *Trypanosoma brucei* (Moraes et al., 2007)*, Trypanosoma cruzi* (Tonelli et al., 2011), *Leishmania* species (Cloutier et al., 2012). Furthermore, three kinases that could potentially phosphorylate eIF2α have been identified (Moraes et al., 2007). In *T. cruzi* these kinases are called *Tc*k1, *Tc*k2, and *Tc*k3. *Tc*k2, which has a topology similar to PERK, had been previously characterized and found in the endosomal compartment of *T. cruzi* (da Silva Augusto et al., 2015), as the homolog of *T. brucei* (Moraes et al., 2007). *Tc*K2 was found to be activated by heme deprivation and gene knockouts showed decreased growth and impaired infectious capacity. The K2 homolog in *Leishmania infantum* is in the endoplasmic reticulum and is required for the intracellular amastigotes' growth (Chow et al., 2011). *Tc*K3 has not yet been characterized, but its ortholog in *T. brucei* has been described and shown to be related to programmed stress-induced cell death (Goldshmidt et al., 2010) by inducing the TATA binding protein phosphorylation (Hope et al., 2014). In *Leishmania donovani*, the K1 (*Lde*K1) was demonstrated to phosphorylate eIF2α under starvation (Rao et al., 2016). The substantial importance of GCN2 homologs in phosphorylating eIF2α and modulating the stress response has also been demonstrated in other protozoan parasites. In *P. falciparum* the GCN2 ortholog was implicated in managing the proper response during amino acid starvation (Fennell et al., 2009) but surprisingly this kinase is not required for the parasite to enter in the hibernation mode during nutritional stress (Babbitt et al., 2012). In *T. gondii*, the GCN2-like ortholog enables extracellular viability and knockouts parasites present fitness defects (Konrad et al., 2011). It was recently shown that upon arginine deprivation, GCN2 induces eIF2α phosphorylation and upregulation of arginine transporters (Augusto et al., 2019). All these studies establish GCN2 at the center of the nutritional stress response in these parasites, modulating a myriad of pathways to avoid cell death, but its role in *T. cruzi* had not yet been established. We hypothesized that *Tc*K1 can also be activated in response to nutritional stress and amino acid deficiency. To test this, we generated *Tc*K1 depleted parasites by gene replacement using CRISPR/Cas9 tools and evaluated its role in nutritional stress responses and importance for its life cycle progression.

## Materials and Methods

The procedures used in this work were approved by the “Comite de Ética em Pesquisa da Universidade Federal de São Paulo” under protocol 266629/2015. All methods were performed in accordance with the relevant guidelines and regulations approved by the Universidade Federal de São Paulo that follows the Brazilian Animal Practice and Ethics legislation.

### Primer Design

The gene sequences were taken from the TriTrypDB website [http://tritrypdb.org] (Aslett et al., 2010). Single guide RNAs (sgRNA) were obtained by *in vitro* transcription as described (Peng et al., 2014). Briefly, the oligonucleotide TcK1-Primer sgRNA48 and sgRNAThr169/2 were designed to contain the T7 RNA polymerase promoter followed by the proto-spacer region of 20 nucleotides and the Cas9 PAM site. These sites were chosen based on the EuPATGDT tool (http://grna.ctegd.uga.edu/) to present the best combination of score, absence of off-targets and the best location for mutation insertion (Table S1). To generate the sgRNA template, the oligonucleotides and a common reverse primer were employed to amplify by PCR a portion of the pUC-sgRNA template plasmid (Lander et al., 2015). The blasticidin resistance DNA sequence flanked by homologous regions to the TcK1 gene was used as DNA repair donor. For the donor production the forward primer (TcK1-Primer Forward for the donor) was designed containing 80 nucleotides corresponding to the 5′ sequence immediately upstream the Tck1 ATG start codon plus 20 initial nucleotides of blasticidin coding region (BSD) from plasmid pTrex-b-NLS-hSpCas9 (a gift from Rick Tarleton obtained from Addgene, plasmid # 62543). The reverse primer (TcK1-Primer Reverse for the donor**)** contained the 20 final nucleotides of the BSD sequence and 80 nucleotides homologous to the sequence of the TcK1 gene downstream to the PAM site (Lander et al., 2015). The donor for the eIF2α T169A mutant was the oligonucleotide eIF2Donnorv2, which contained an indicator BssHII restriction site. The total DNA of the generated eIF2α T169A mutant parasites were amplified by PCR using primers to eIF2α and the product digested with BssHII and the correct substitution confirmed by DNA sequencing.

### *In vitro* Transcription for sgRNA Generation and Donor DNA Generation

For *in vitro* transcription, the sgRNA template generated by PCR was used as scaffold for the reactions using the MEGAShortscript Kit (Thermo Fisher Scientific) according to the manufacturer instructions. After the transcription reactions, the RNA was extracted with 1:1 phenol/chloroform and recovered by precipitation overnight at −20°C by the addition of 100% ethanol and ammonium sulfate to 0.3 M. The samples were centrifuged at 13,000 g for 15 min at 4°C, the supernatant was discarded, and the RNA pellet was resuspended in 20 μL of RNAse free water and stored at −80°C. This final product was used later as sgRNA for transfection of parasites. To produce the donor for TcK1, a conventional PCR was performed using the TcK1-Primer Forward for the donor and TcK-Pirmer Reverse for the donor to amplify the blasticidin (BSD) coding region from the plasmid pTREX-b-NLS-hSpCas9 (Lander et al., 2015). The donor for the eIF2α T169A mutant (eIF2Donnorrv2) was chemically synthesized.

### Generation of Mutated Parasites

*Trypanosoma cruzi* wild type Y epimastigotes were cultured in medium containing liver and infusion tryptose (LIT) supplemented with 10% fetal bovine serum at 28°C. The parasites expressing Cas9 were generated by transfection with plasmid pTREX-Cas9-Neo (Lander et al., 2015), selected and maintained in the same medium supplemented with 200 μg/mL Geneticin G418. For transfection, 4 × 10^7^ exponentially growing Y-Cas9 epimastigotes were collected and centrifuged at 2,000 g for 5 min, parasites were washed in 1 mL of electroporation buffer (5 mM KCl, 0.15 mM CaCl_2_, 90 mM Na_2_HPO_4_, 50 mM HEPES, 50 mM Mannitol) (Pacheco-Lugo et al., 2017) and resuspended in 100 μL of electroporation buffer, along with 50 μL containing 2 μg of purified sgRNA and 25 μg of purified PCR fragments containing the BSD sequence. Parasites were transfected in 2 mm cuvettes using AMAXA Nucleofactor II apparatus, with two pulses of X-014 program. Subsequently, parasites were transferred to bottles with fresh LIT medium supplemented with 10% fetal bovine serum and maintained at 28°C for recovery. After 18 h, BSD (100 μg/mL) was added for selection of TcK1 knockout parasites. After transfection and recovery time, cultures were evaluated by PCR genotyping to confirm the insertion of the desired resistance marker and TcK1 gene disruption. PCRs of the samples to determine the presence of the TcK1 gene were made using the primers 5K1fowApal (P1) and TcK1KDRevXho (P2) and the correct insertion of BSD with primers BSDFow (P3) and BSDRev (P4). To test for the elimination of TcK1 ORF, we used the primers CDSK1FowNdel (P5) and 1TcK1KDRevXhoI (P2). For the generation of eIF2α in which the threonine 169 was replaced by alanine, the same transfection procedure was used, but without antibiotic selection, as the donor was the oligonucleotide eIF2Donnorv2.

### Western Blot

Parasites were collected by centrifugation (2,000 × g for 5 min), washed in PBS and resuspended in TDB buffer [5 mM KCl, 80 mM NaCl, 1 mM MgSO_4_, 20 mM Na_2_HPO_4_, 2 mM NaH_2_PO_4_, 20 mM glucose (pH 7.4) containing EDTA-free Complete-C protease inhibitor and PhoSTOP (Sigma-Aldrich)] and lysed by three freeze and thaw cycles in liquid nitrogen and at 37°C. For the differential solubilization experiments, 5 X 10^7^ washed parasites were resuspended in 100 mL of 50 mM Tris-HCl (pH 7.6), 50 mM NaCl, 5 mM MgCl_2_, 0.1% Nonidet-P40, 1 mM β-mercaptoethanol, EDTA-free Complete-C protease inhibitors at 4°C. After 10 min, the lysates were centrifuged 10 min at 10,000 g and the supernatant, corresponding to the soluble fraction transferred to a new tube. The insoluble precipitate was then resuspended in 100 μL of the same buffer. For both types of lysates 5XSDS-sample buffer was added and the samples boiled for 5 min before fractionation by 10% SDS-PAGE and transference to nitrocellulose membranes. After transfer, membranes were incubated in 10 mM Tris-HCl, pH 7.4, 0.15 M NaCl (TBS) containing 5% BSA and 0.05% Tween-20 for 2 h for blocking and then incubated 2–12 h in the same buffer with the primary antibodies. After three washes of 10 min each with TBS containing 0.05% Tween-20, bound antibodies were detected by 1-h incubation in washing buffer with the α-rabbit or α-mouse IgG coupled to IRDye 800 and IRDye 680 (LI-COR Biosciences) antibodies, respectively, three 10-min washes, and acquisition using an Odyssey Fc System (LI-COR Biosciences). Rabbit antibodies directed to *T. cruzi* eIF2α phosphorylated at Thr 169 were generated as previously described (Tonelli et al., 2011). Specific antibodies were re-purified by sequential affinity chromatography on peptides NH_3_YTEI[^P^T]RIRIRAIGKC-amide and NH_3_YTEIRIRIRAIGKC-amide (GeneScript) both coupled to SulfoLink^®^ Coupling Resin (Thermo Fisher Scientific). Elution from the first column was achieved by using 50 mM glycine pH 2.8 buffer, followed by neutralization by adding 2 M Tris-HCl pH 9.0 and the second column was only used to adsorb non-phospho-specific antibodies. α-*T. cruzi* eIF2α antibodies that recognize the full protein was obtained by immunizing mice with the *T. cruzi* recombinant protein cloned in pET28a and expressed in *E. coli* BL21 as described previously (da Silva Augusto et al., 2015). Antibodies to the *T. cruzi* PABP1 and *T. brucei* biphosphate aldolase proteins were generated by immunizing rabbits with the respective recombinant proteins cloned in pET28a, expressed in *E. coli* and purified in Ni^2+^-agarose columns. α-PABP1 antibodies were further purified by adsorption on the recombinant protein transferred to nitrocellulose membranes. α-tubulin was obtained from Sigma-Aldrich (T-6074).

### Immunofluorescence

*Trypanosoma cruzi* cultures at log growth phase (0.8 and 1 × 10^7^ cells/mL) were used for the immunofluorescence assays. Parasite cultures were centrifuged, washed twice in PBS and then resuspended in 4% PFA in PBS. After 30 min, the cells were washed in PBS twice and then resuspended in PBS to 5 × 10^4^ cells/mL. About 10 μl of the sample was distributed into an 8-well slide and allowed to dry at room temperature. The cells were then rehydrated in PBS and permeabilized for 5 min in PBS containing 0.1% Triton X-100, washed twice and incubated for 1 h at room temperature with blocking solution (5% BSA in PBS). After blocking, the slides were incubated with the primary α-DHH1 antibody diluted 1: 100 in blocking solution. The antibodies were prepared in mice by using the recombinant protein generated as described (Costa et al., 2018). The serum was precipitated with 30% ammonium sulfate and resuspended in the original volume in PBS. After three consecutive washes with PBS-T (PBS with 0.1% Tween 20) the slides were incubated 1 h with the mouse α-IgG Alexa 594 (Thermo Fisher Scientific) with 20 μg/mL of Hoechst DNA marker for 1 h. Following three washes in PBS-T the slides were mounted, and images were taken using ×100 plan Apo-oil objective (NA 1.4) in an Olympus (BX-61) microscope equipped with a Hamamatsu Orca R2 CCD camera. Acquisitions were made through the Z-axis in 0.2 μm sections. Image analyses were done using Autoquant 2.2 (Media Cybernetics). For quantification of the DHH1 granules after immunofluorescence, the parasites from several photos were taken in randomly chosen fields at 100x magnification and in different focal planes for Z series composition. The total number of granules was manually quantified at the maximum projection for a least 100 parasites of each strain.

### Polysome Profile

The polysome profile was generated using the method previously described (Holetz et al., 2007). Briefly, cycloheximide (100 μg/mL) was added to exponentially growing parasites (1 × 10^9^) and cells were kept at 28°C for 10 min before incubation on ice for another 10 min. For analysis of parasites subjected to nutritional stress, the same number of parasites was incubated in Triatoma Artificial Urine medium [TAU, 0.19 M NaCl, 0.017 M KCl, 2 mM CaCl_2_, 2 mM MgCl_2_, 8 mM potassium phosphate (pH 6.8)] for 1 h at a concentration of 10^9^ parasites/mL prior to treatment with the same proportion of cycloheximide (200 μg/10^7^ parasites). Afterwards, the parasites were centrifuged for 5 min at 2,000 g at 4°C, washed in ice-cold PBS containing 100 μg/mL cycloheximide and resuspended in 450 μL of TKM buffer (10 mM Tris-HCl, pH 7.4, 300 mM KCl, 10 mM MgCl_2_ and 100 μg/mL cycloheximide) containing 10 μM E-64, 1 mM PMSF, 0.2 mg/mL heparin.

Parasites were lysed by adding 50 μl of 10% NP-40, 2 M sucrose in TKM buffer by gentle mixing. The lysates were centrifuged for 5 min at 10,000 g at 4°C and the supernatant was transferred to the top of a previously prepared sucrose gradient from 15 to 55% sucrose in TKM buffer using a gradient generator (Gradient Master, Biocomp). Samples were ultracentrifuged at 39,000 r.p.m. for 3:00 h in a Beckman SW41 rotor at 4°C and fractions collected and analyzed by continuous injection of 62% sucrose at 0.6 mL/minute using the Econo Gradient Pump (Bio-Rad) followed by absorbance detector analysis at 254 nm.

### Metacyclogenesis

For epimastigote differentiation, parasites were washed and resuspended in TAU medium to 1 × 10^9^ parasites/mL and incubated for 1 h at 28°C. After this initial incubation period, the parasites were diluted 100x in TAU medium containing 10 mM glucose, 2 mM L-aspartic acid, 50 mM L-glutamic acid and 10 mM L-proline (TAU3AAG) and 10 mL incubated in laid down 25 cm^2^ tissue culture flasks at 28°C for 5 days. Alternatively, 1 volume of epimastigotes exponentially growing cultures (2–4 × 10^7^/mL) were incubated with 3 volumes of Grace's Insect Media (Thermo Fisher Scientific) at 28°C for 5 days. After the differentiation period, the number of parasites was counted by using a Neubauer chamber and the differentiation rate assessed by staining with Giemsa solution. Aliquots of the differentiated parasites were also used for cell infection experiments to obtain tissue culture trypomastigote forms.

### Parasite Infection

The trypomastigotes were maintained in human U-2 OS osteosarcoma cells (ATCC) plated a day before infection in 75 cm^2^ flask incubated at 37°C in low glucose DMEM (Thermo-Fischer Scientific) supplemented with 10% fetal bovine serum (SFB), 59 mg/mL penicillin and 133 mg/mL streptomycin in a CO_2_ incubator. The cells were initially infected with cultures incubated with DEAE-cellulose to obtain purified metacyclic-trypomastigotes (Yoshida, 1983). For the next round of infections, we used tissue culture-derived trypomastigotes (TCTs) released from the infected cells. To quantify parasite infection, we performed infections of 3 × 10^4^ U-2 OS cells seeded in 96-well plates containing 100 μL of medium. The next day, 1.5 × 10^6^ trypomastigotes in 50 μL of medium corresponding to a multiplicity of infection of 25 TCTs per cell were added and plates incubated for 6 h at 37°C. Afterwards, the media were removed, wells were washed with PBS and fresh medium was added. The plates were incubated for 72 h in a CO_2_ incubator for better detection of the amastigotes nests and after this period, the cells were fixed with 4% paraformaldehyde in PBS and stained with Draq5 (Biostatus). Then, the plates were scanned using a High Content Imaging System In Cell Analyzer 2200 (GE) at 20X magnification and the acquired images analyzed by using the In Cell Investigator Software 1.6 (GE). Five images of each well were acquired and analyzed for determination of the infection ratio. Three different sets of infections were performed, enabling the analyses of thousands of cells. Non-infected cells were used as negative controls.

### Sequence and Statistical Analyses

Sequences were analyzed through blast, and after *in silico* translation of the predicted open reading frame, the domains were identified by Pfam (El-Gebali et al., 2019) and HHpred Analyses (Zimmermann et al., 2018). Multiple sequence alignment was generated by the Muscle method (Edgar, 2004). For the phylogenetic analysis, the sequences were downloaded from TriTryp.org and the translated sequences corresponding to the kinase domain of the proteins aligned by 8 iterations using the MUSCLE program included in the Geneious 11.1.15 software. Phylogenetic trees were generated by PHYML also included in the Geneious package. Graphic and statistical analysis were performed using Prism 7 (GraphPad).

## Results

### *In silico* Identification of *T. cruzi* GCN2

**Figure 1:**
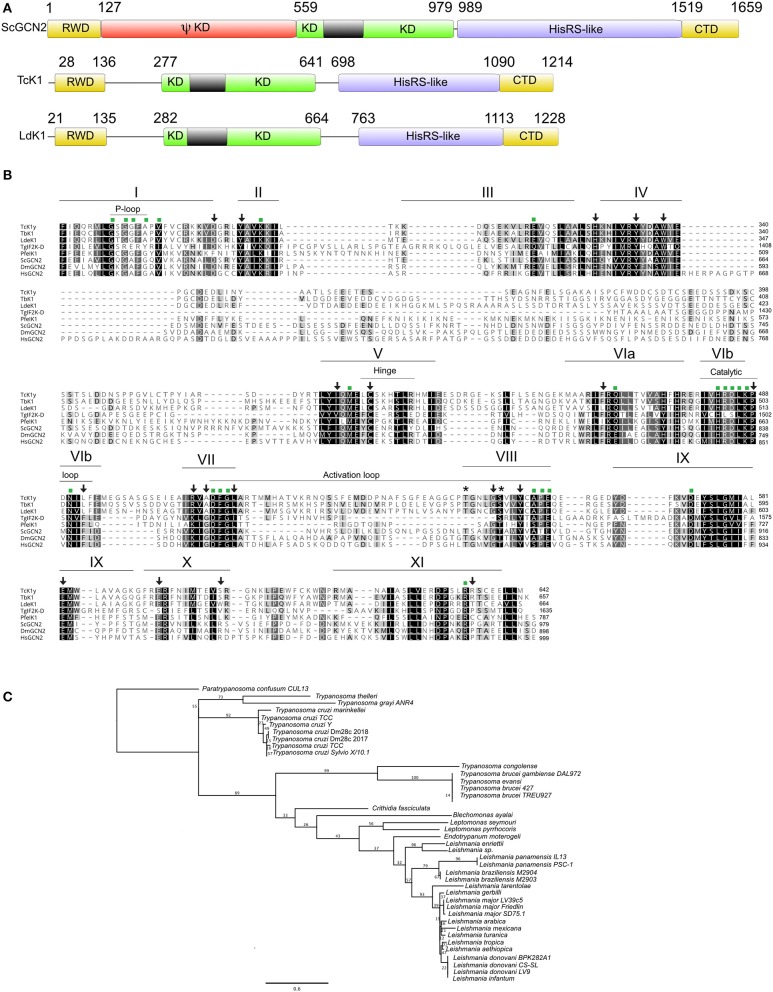
**(A)** Schematic representation of Tck1 gene sequence with predicted domains in comparison with the *Saccharomyces cerevisiae* (ScGCN2) and *Leishmania donovani* (LdeK1). Pfam analysis predicted the RWD and kinase domain in Tck1 gene in the N-terminal portion and HHpred analysis identified the HisRS-like domain despite its low sequence conservation. The black boxes indicate the typical insertion in the kinase domain of eIF2α kinases. The panel also indicates CTD domain, and the pseudo-kinase (ΨKD). The numbers indicate the position of each domain. **(B)** Sequence alignment of the kinase domain of *Tc*K1 in comparison with GCN2 of model organisms that have been already characterized. Identical residues are indicated in black shades and similar residues are in light gray. The bars on the top with roman numerals correspond to the eleven subdomains characteristic of the GCN2 kinases. Asterisks indicates the residues that undergo auto phosphorylation in eIF2α kinases in the VIII subdomain. Green squares indicate residues conserved in Ser/Thr protein kinases and arrows the residues maintained in eIF2α protein kinases. The positions of the P-loop, hinge, catalytic loop and activation loop are indicated by thin bars. The sequence for the *Tc*K1 from the Y strain was obtained from Tritryp.org from the nucleotide sequence NMZO01000706.1:2,035.5,676. The other sequences were downloaded from GenBank with the following accession numbers: *Toxoplasma gondii*: AED01979.1; *T. brucei*: XP_828792.1; *Saccharomyces cerevisiae*: DAA12123.1; *Plasmodium falciparum*: XP_001348597.1; *Leishmania donovani*: AKG62099.1; *Homo sapiens*: Q9P2K8.3; *Drosophila melanogaster*: AGB96521.1 **(C)** Phylogenetic tree of the amino acid sequences corresponding to the kinase domain of the homologous of GCN2 of the indicated Kinetoplastidae species. The tree was generated with PHYML using the LG substitution model and 100 bootstraps (Guindon et al., 2010). The access numbers for these sequences are shown in Table S2.

The prediction of the *Tc*K1 protein comprises the relevant domains for the GCN2 signature (Figure 1A). Pfam analysis identified the RWD (RING finger-containing proteins, WD-repeat-containing proteins, and yeast DEAD (DEXD)-like helicases) and kinase domains. The RWD domain was found located in the N-terminal portion (AA^28−136^). In mammals and yeast this sequence binds to GCN1 regulatory protein being important for GCN2 full activation (Dever and Hinnebusch, 2005; Castilho et al., 2014). As was reported for *L. donovani* (Rao et al., 2016), *P. falciparum* (Fennell et al., 2009), and *T. gondii* (Konrad et al., 2014), *Tc*K1 also lacks the pseudo-kinase domain. HHpred analysis identified another hallmark characteristic in the *Tc*k1 sequence, the presence of the HisRS-like domain (AA^698−1090^) in the C-terminal portion (CTD), despite its low level of sequence conservation. This domain resembles the His-tRNA synthetase domain but lacks significant residues to be enzymatically active although it was shown to bind to uncharged tRNA and to act during GCN2 activation (Wek et al., 1995). The kinase domain (KD) (AA^277−641^) includes all of the eleven subdomains characteristic of protein kinases (Hanks and Hunter, 1995; Olsen et al., 1998) and the typical eIF2α kinase insertion (denoted by the gray box), which varies in size in different organisms (Ramirez et al., 1992) (Figure 1B). This insertion presents little conservation even among the trypanosomatids analyzed (8.3% identical sites). Little is known about the role of this insertion in the kinase domain, although works have demonstrated its importance for heme binding and activation of HRI (Rafie-Kolpin et al., 2000; Pakos-Zebrucka et al., 2016). The insert was also found to increase the binding of PERK to the eIF2 substrate (Marciniak et al., 2006). Most of the conserved residues among general protein kinases (Hanks and Hunter, 1995), indicated by green squares in Figure 1B, are identical or highly similar, with exception of the Ala^287^ that replaces the conserved Gly residue in the Gly-rich P-loop at the ATP binding motif in *T. cruzi* as in the other trypanosomatids. There are, however, subtle differences in residues more specific for eIF2α protein kinases (Padyana et al., 2005), indicated by arrows in Figure 1B, specifically the L492F and A510G substitutions, also found in other trypanosomatids. Nevertheless, other substitutions such as S617R and R635P are not conserved in any of the protozoa parasites. Actually, the subdomains IX-XI present a lower level of conservation in kinases and seem to be less important for kinase activity. It is also important to highlight the conservation of the Thr^548^ and Ser^553^ residues, the latter of which is substituted to a threonine in yeast, *Drosophila* and mammals. These residues correspond to one of the two threonine residues involved in the autophosphorylation of PKR in the activation loop, which is relevant for substrate recognition (Romano et al., 1998; Dey et al., 2005).

A phylogenetic tree based on the amino acid sequence of the KD indicates a clear separation of at least three distinct groups of GCN2 in Trypanosomatids following the expected speciation based on ribosomal small subunit analysis, with the most distant species being *Paratrypanosoma confusum* (Figure 1C). All *Leishmania* species forms one of the three groups. Although presenting very few amino acid substitutions, the separation of the subgenus Leishmania from Viannia can be detected. The tree also correlates quite well with the separation between the *T. cruzi* and *T. brucei* clades. Interestingly, the inserts in the KD were conserved among each species group, suggesting possible differences between the enzymatic function in the parasites (Figure S1). However, we found no evidence of episodic diversifying selection in the phylogeny considering the insert in the KD of all Kinetoplastidae species sequenced so far by using adaptive branch-site REL test for episodic diversification (aBSREL, http://datamonkey.org/absrel), suggesting that the differences observed in each species might represent a random event during the evolution. In *T. cruzi* for example, the inserts, although conserved, contain 3 silent mutations in the third base and 4 non-silent mutations.

### Generation of *T. cruzi* Depleted From TcK1 Gene

**Figure 2:**
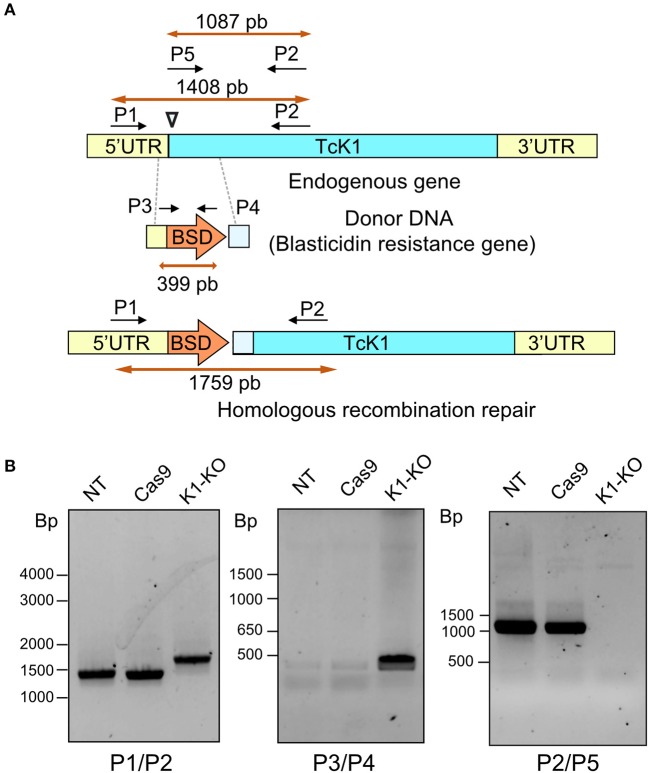
**(A)** Illustrative diagram of the approach used to generate TcK1 knockout. A PAM site (inverted triangle) was chosen in the begin of the Tck1 ORF (blue) (Top). The donor DNA sequence was prepared comprising the BSD resistance gene and homologous sequences to the Tck1 5′UTR (yellow) and to the ORF immediately after the PAM site (light blue) to favor the repair by homologous recombination (Middle). The bottom diagram denotes the correct integration of the donor DNA to generate the TcK1 interrupted ORF. P1 and P2 represent the pair of primers used to amplify the TcK1 gene corresponding to the N-terminal sequence of the protein and P3 and P4 primers used to amplify the BSD sequence. P5 indicates the position of the primer that aligns with the removed region of TcK1 gene. **(B)** *T. cruzi* genomic DNA was isolated and genotyped by PCR using a combination of primers to amplify the endogenous genes of TcK1 (P1 and P2), the BSD integration (P3 and P4) and to detect the original ORF of TcK1 (P5 and P2). The numbers on the left of each gel stained with ethidium bromide represent the migration of DNA size markers.

To better understand the role of *Tc*K1 in *T. cruzi*, we generated parasites in which the *Tc*K1 was interrupted by insertion of the BSD resistance encoding gene by using the CRISPR/Cas9 system (Figure 2A). In this approach, the donor DNA was obtained by PCR using as a template a BSD resistance gene flanked by sequences homologous to the TcK1 gene. The donor DNA was generated by PCR and inserted into epimastigotes parasites expressing the Cas9 enzyme, already resistant to geneticin G418 together with the sgRNA, which was synthesized by *in vitro* transcription. We obtained parasites resistant to G418 and BSD 3 weeks after transfection. Expected insertion of BSD disrupting the targeted gene was verified by PCR using primers P1 and P2. It was possible to verify that the band size increased for the TcK1 gene locus, compatible with the insertion event (Figure 2B). The presence of the correct insertion of the BSD and absence of the TcK1 ORF were also evaluated by PCR using primers P3/P4 and P1/P2, respectively (Figure 2B).

### Parasites Depleted of TcK1 Gene Show Increased Polysome Levels

**Figure 3:**
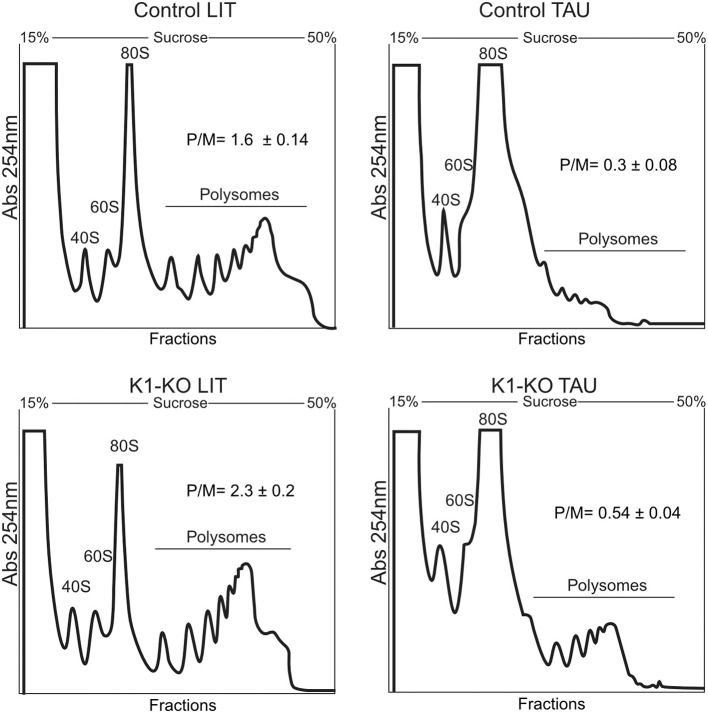
Polysome profile analysis of control and TcK1 depleted parasites (*Tc*K1-KO). Epimastigotes were cultivated in LIT medium and incubated in LIT or in TAU medium for 1 h. The parasites extracts were prepared as described in Methods and fractionated by ultracentrifugation through a 15–55% sucrose gradient. The fractions were collected from the top to bottom and the amount of RNA estimated by the absorbance at 254 nm. In each panel it is indicated the migrating position of the 40S, 60S, 80S and the polysomal fractions. The amount of polysomes relative to the 80S (P/M) was estimated by measuring the area under each peak in triplicate samples.

We found that epimastigotes parasites lacking TcK1 gene showed similar growth rates compared to control lineages when maintained under standard culture conditions. Apparently, neither the replication capacity nor the morphology was affected in the epimastigotes (see 3.5 and 3.6 topics). Considering the role of *Tc*K1 in performing eIF2α phosphorylation during nutritional starvation and the possible inhibition of translation initiation stage, we performed experiments to analyze the polysome profile of the TcK1 depleted strain in normal and in starved parasites. The experiments were performed using samples prepared from control and mutated parasites grown in LIT medium compared to both parasite lines subjected to incubation in TAU medium for 1 h. We observed that the polysome amounts, particularly the ones containing more than 4 ribosomal subunits were consistently enriched in the TcK1 depleted line compared to the control line (Figure 3). Upon starvation, the levels of polysome decreased 5 times in the control line and 4 times in the TcK1-KO. These results suggest that GCN2 homolog in *T. cruzi* could be involved in the translation control.

### Parasites With Mutated *Tc*K1 Are Still Able to Phosphorylate eIF2α Under Nutrient Deprivation

**Figure 4:**
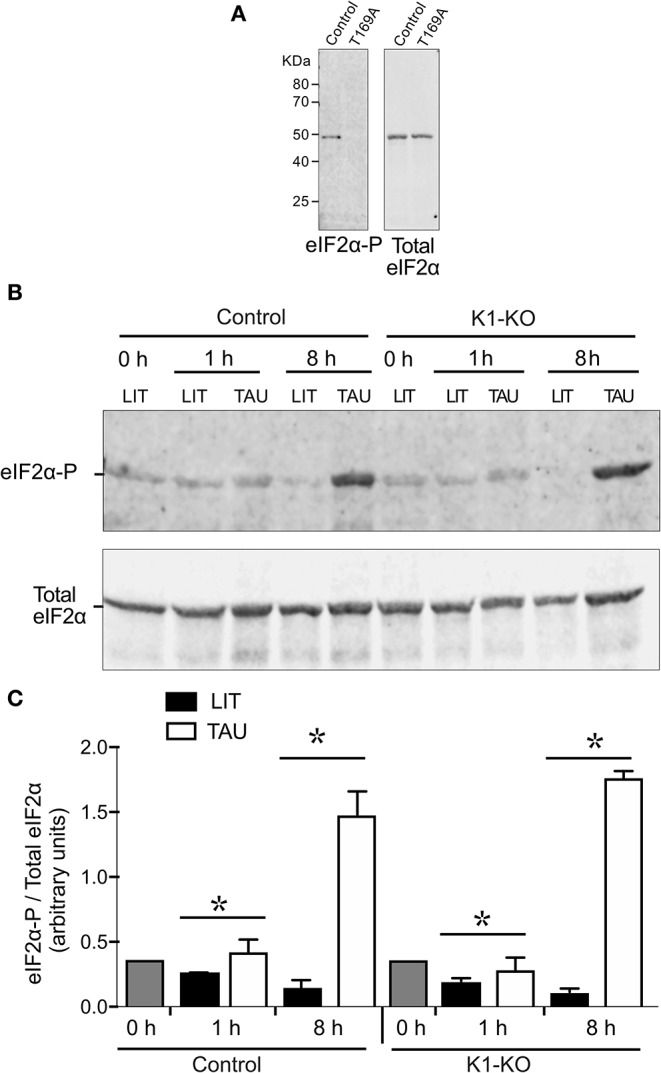
Western blot analysis reveals that TcK1-KO parasites are still able to phosphorylate eIF2α. **(A)** Extracts of control epimastigotes and eIF2 mutants (T169A), were obtained from cultures at 1 × 10^7^ in LIT medium, lysed as described in Methods and the equivalent of 3 × 10^6^ parasites loaded per lane. The Western blot was revealed after incubation with the affinity-purified rabbit α-phospho-eIF2α and revealed with α-rabbit IgG-IRDye-680. After imaging, the membranes were probed with mouse α-total *T. cruzi* eIF2α. **(B)** Exponentially growing epimastigotes were incubated at LIT or TAU medium for the indicated periods of time, and processed as in **(A)**. The figure shows one of three experiment. **(C)** Relative ratios of phosphorylated eIF2α and total eIF2α labeling (means ± S.D, *n* = 3). Asterix indicate significant differences (*p* < 0.05) using two-way Anova.

To gain more information about the role of *Tc*K1, we examined the levels of eIF2α phosphorylation after nutritional stress in both control and mutated parasites. For that, we used an α-phospho-eIF2α labeling that was specific for the phosphorylated protein at threonine 169, as demonstrated in the *T. cruzi* line expressing a mutated version of eIF2α in which the threonine 169 was replaced by alanine showed no labeling (Figure 4A). Surprisingly, both cell lines showed a progressive increased phosphorylation levels of eIF2α after incubation in TAU medium from 1 to 8 h (Figure 4B). Interestingly, in this experiment, we noticed that the eIF2α phosphorylation diminished progressively in rich medium when the growing parasites were diluted in fresh medium in both control and mutated cells, with a more pronounced decrease in the TcK1-KO cells (see LIT 8 h points), possibly explaining the increased amounts of polysomes in these cells. A quantitative analysis of phosphorylation is shown in Figure 4C. This finding suggests that other eIF2α kinases are able to phosphorylate eIF2α under nutritional depletion, mainly at longer incubation periods. In addition, *Tc*K1 seems to affect the low level of phosphorylation in rich medium.

### TcK1 Depleted Line Show Reduction of Stress Granules Formation Under Starvation

**Figure 5:**
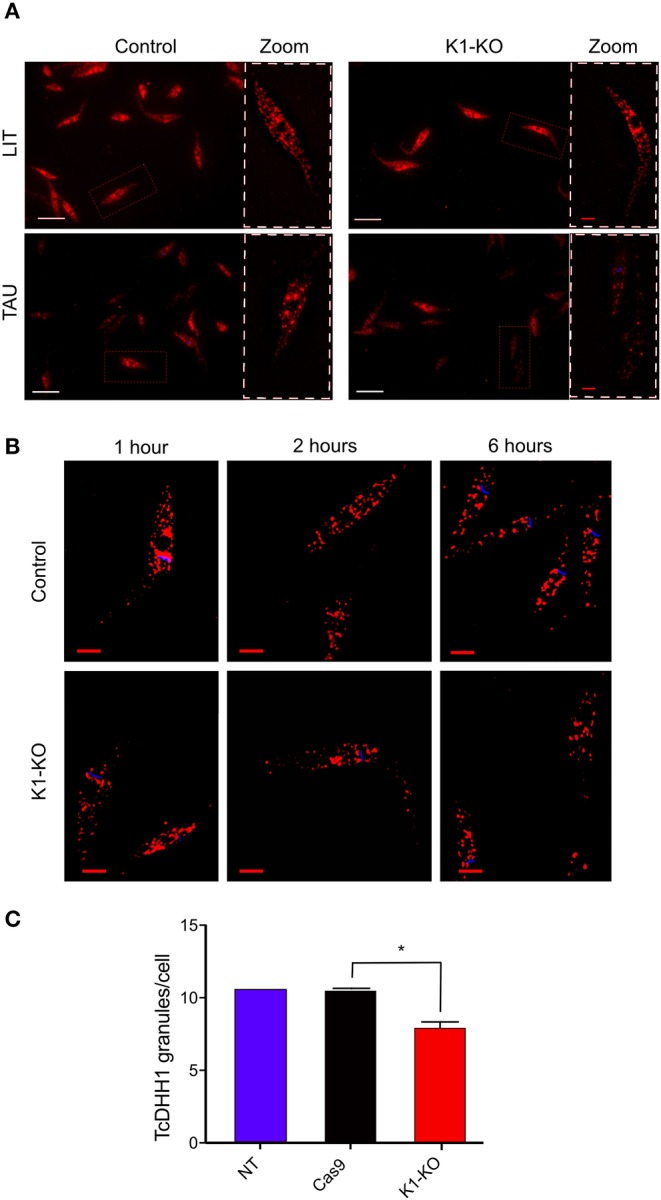
*Tc*K1 depleted parasites show less DHH1-ribonucleoprotein granules upon nutritional stress than control parasites. **(A)** Representative images of DHH1 immunofluorescence (red) and Hoechst dye staining (blue) of control and *Tc*K1-KO parasites maintained in LIT medium or after incubation in TAU medium for 6 h. The zoomed images correspond to the region indicated by white doted lines. Bars = 2 μm. **(B)** Enlarged z-sections of the same parasites incubated for 1, 2, and 6 h in TAU medium. Bars = 2 μm. **(C)** Quantification of the average number of large granules per cell in epimastigotes incubated in TAU medium for 6 h. The numbers were the mean ± SD (*n* = 100). The Asterisks indicate a significant difference (*p* < 0.05), using two-way Anova test.

It has been found that mRNAs can be stored in granules containing RNA binding proteins in unfavorable scenarios such as during nutrient scarcity (Guzikowski et al., 2019). At the same time, translation is inhibited. Thus, taking into account this context, and the fact that GCN2 is involved in stress granule formation (Panas et al., 2016), we investigated the formation of RNA granules in *Tc*K1-KO line during nutritional stress through immunofluorescence. As a marker for granule formation, we used the antibody against DHH1 protein, a protein already well-described as part of the P-body granules (processing bodies) in higher eukaryotes (Aulas et al., 2017; Guzikowski et al., 2019). DHH1 has also been identified in trypanosomatids as part of granules (Kramer et al., 2008, 2010; Holetz et al., 2010). In Y strain epimastigotes, DHH1 labeled small dots distributed over the entire cytosol in both control and in mutated *Tc*K1 (Figure 5A). The labeling excluded the nucleus and the mitochondrion, as well as other intracellular organelles. After incubation for 1, 2, and 6 h in TAU medium, the labeling appeared progressively more intense in discrete regions of the cytosol in control cells. Within 1 h of nutritional stress, α-DHH1 labeling detected very small granules located in the perinuclear area with a small decrease in the cytoplasmic signal. Within 6 h, all cells showed large granules, concomitant with decreased cytoplasmic signal (Figure 5B). This pattern was not so evident in *Tc*K1-KO parasites, in which <10% of the cells present evident granules in the first h. Therefore, we performed a quantitative analysis 6 h after the stress, which showed a significant decrease in the number of granules in the K1-mutated parasites (Figure 5C). To remove small granules in these analyses the images were modified to include threshold that clearly showed a decrease in the large granules in the line in which *Tc*K1 was modified (Figure S2). Importantly, the number of granules identified was very similar in the non-transfected Y and Cas9 transfected parasites. These results suggest that *Tc*K1 depletion disturbed DHH1 granule formation in *T. cruzi*.

**Figure 6:**
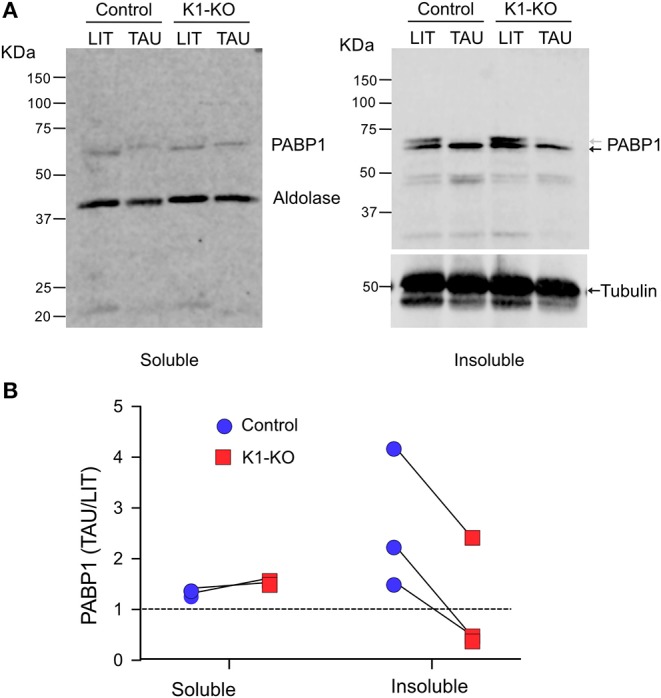
*Tc*K1 depleted parasites show reduced enrichment of PABP1 in the insoluble fraction upon nutritional stress than control parasites. **(A)** Western blot of the soluble and insoluble fractions of extracts corresponding to 1 × 10^7^ epimastigotes prepared as described in methods from parasites incubated for 6 h in LIT or TAU medium. The gels were probed with rabbit α-PABP1 and α-aldolase, and mouse anti-α-tubulin. **(B)** Relative enrichment of PABP1 compared to aldolase or α-tubulin to the soluble and insoluble fractions of control (blue) and *Tc*K1-KO parasites (red) in three different experiments.

Stress granules are large ribonucleoprotein complex that include proteins involved in translation initiation as eukaryotic initiation factors 4E, 4G and 3 as well as poly-A binding proteins (Anderson and Kedersha, 2006). The same set of proteins were also found in *T. brucei* RNA granules induced upon stress (Fritz et al., 2015). In addition, these granules contained eIF2 subunits and GCN1, a protein known to regulate the GCN2 (Castilho et al., 2014), the homolog of TcK1. Therefore, to confirm the participation of TcK1 in the stress granule formation in *T. cruzi*, we measured the amount of poly-A binding protein 1 (PABP1) in insoluble materials that contained RNA granules. The measurements were made in wild type and *Tc*K1-KO incubated in control or stress conditions. As shown in Figure 6A, soluble PABP1 levels decreased in parasites incubated in TAU for 6 h in control parasites compared to *Tc*K1-KO in two independent experiments. More important, the expected increase in the granules of control cells incubated in TAU was reduced in *Tc*K1-KO parasites. Similar results were obtained in at least 3 independent experiments (Figure 6B), supporting the notion that the absence of the kinase diminished the stress granules. Interestingly, in both cases, cells presented a double band that disappear after incubation in TAU, which can be related to its phosphorylation state.

### Tck1 Affects Metacyclogenesis and Host Cell Infection

**Figure 7:**
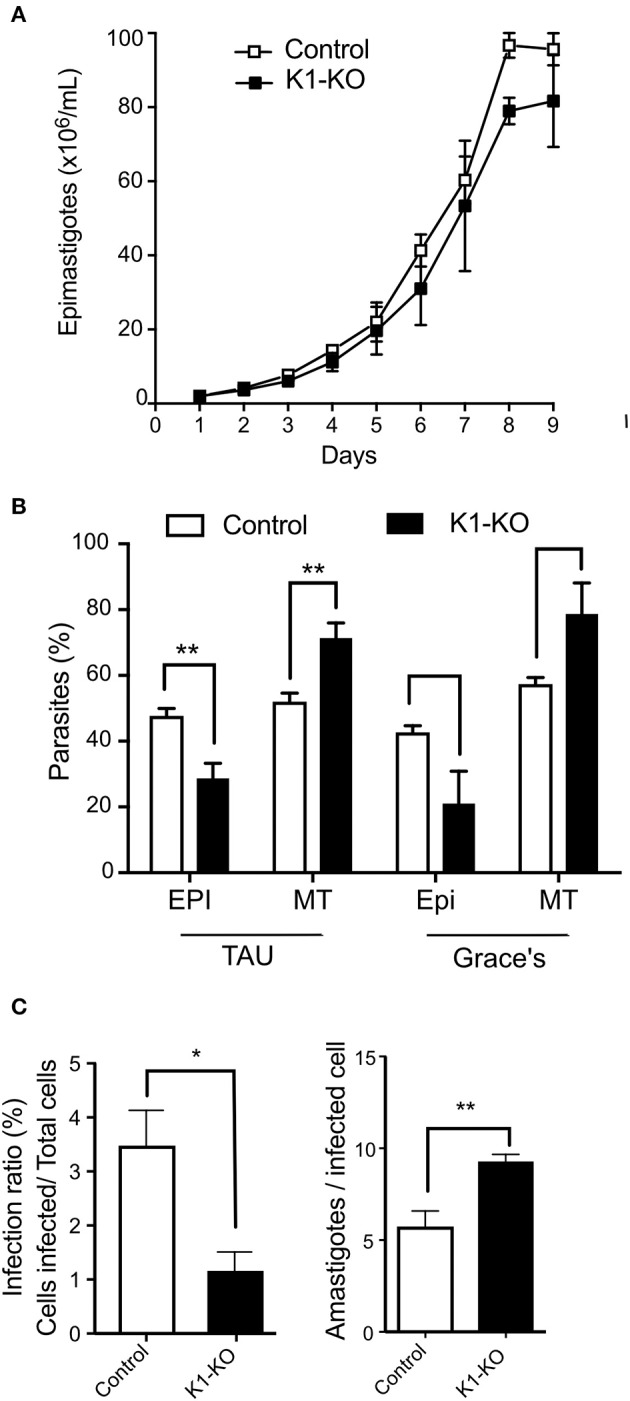
Fitness of *Tc*K1 depleted parasites in the different life cycle stages. **(A)** Parasites of the indicated lines were cultivated in LIT medium, supplemented with 10% SFB. The parasite concentration was measured daily by counting on MUSE equipment and a Neubauer chamber (*n* = 3). **(B)** Epimastigotes of the indicated lines in late exponential growth phase (3 × 10^7^/mL) were collected by centrifugation and incubated in TAU medium at 1 × 10^8^/mL for 1 h. Afterwards, the parasites were then diluted to 5 × 10^6^/mL in TAU 3AAG and maintained for 5 days to allow differentiation into metacyclic forms. Alternatively, the LIT cultures were centrifuged and resuspended to 5 × 10^6^/mL in Grace's medium and incubated for 5 days. At the end of incubation, the cells were stained by Giemsa and the number of metacyclic and epimastigotes forms counted in each case. The graphic shows the mean ± SD of three independent experiments. **(C)** Trypomastigotes of control and *Tc*K1-KO lines (7.5 × 10^5^) in a volume of 0.1 mL obtained from the supernatant of infected cells were used to infect U2-OS cells (3 × 10^4^) seed a day before in 96 wells plates. After 6 h, the parasites were removed, the wells were washed, and one-half of the wells fixed with 4% *p*-formaldehyde in PBS and the other half incubated with fresh medium for 72 h, before washing and fixation. The number of intracellular parasites was then quantified by imaging the fluorescent parasites per cell both labeled with Draq5. The values are means ± SD of three independent experiments, each one corresponding to values of 5 wells. Asterisks indicate *p* < 0.01 (^*^) or 0.05 (^**^) calculated using the Student *t*-test.

Because nutritional stress (Romaniuk et al., 2018), and eIF2α phosphorylation (Tonelli et al., 2011) have previously been associated with parasite differentiation, we investigated the effects of *Tc*K1 absence on parasite metacyclogenesis. As mentioned above, no growth effect was found by comparing control and mutated cell lines (Figure 7A). However, *Tc*K1-KO showed an increased differentiation from epimastigotes to metacyclic-trypomastigotes compared to control parasites when induced to differentiate in TAU medium followed by incubation in TAU3AGG (Figure 7B). Similar results were observed when the differentiation was induced in Grace's medium.

Next, the obtained metacyclics were used to infect mammalian cells and to generate cell-derived trypomastigotes. When trypomastigotes were probed for their infection capacity, we found a large decrease (more than 90%) in the infectivity of the *Tc*K1-KO compared to the control parasites (Figure 7C). Interestingly, the number of obtained trypomastigotes from the infected cells was similar in both cases. Indeed, after 72 h, the number of intracellular parasites was even higher in mutated cell lines, suggesting that although less infective, the cell line lacking *Tc*K1 replicated faster in mammalian cells.

## Discussion

We characterized the *T. cruzi* GCN2 homolog, named *Tc*K1. This protein kinase was found highly conserved through different Kinetoplastidae. We found that *Tc*K1 presents the main characteristics of eIF2α protein kinases. The most divergent region was the insertion of the kinase domain, postulated to make a loop involved in the substrate recognition. Nevertheless, these inserts were conserved in Kinetoplastidae species and our phylogenetic analysis indicated a non-selective pattern in each case, suggesting a random evolution, not necessarily related to the enzymatic role, or substrate recognition.

Through genome editing with CRISPR/Cas9, we provided evidence that *Tc*K1 is not the main protein kinase involved in the eIF2α phosphorylation under nutrient deprivation, differently to what has been shown in many organisms, including *L. donovani* (Rao et al., 2016). Nevertheless, the absence of *Tc*K1 decreased the formation of DHH1-containing granules under nutritional stress, possibly related to the action through a different mechanism, as shown for translation arrest induced by TIA-1 and TIAR proteins in human cells (Damgaard and Lykke-Andersen, 2011). At the same time, less insoluble PABP1 was found when parasites were incubated under nutritional stress. Finally, we found that in absence of TcK1 the capacity of parasite differentiation, mammalian cell invasion and proliferation in the host was largely affected, all suggesting that TcK1 modifies the expression of proteins relevant at the different moments of the parasite life cycle.

We depleted the TcK1 gene by the insertion of the BSD sequence at the beginning of the TcK1 gene. The same drug resistance approach has been used to generated gene depletions in other Trypanosomatids (Lander et al., 2015; Beneke et al., 2017). In our case, the correct insertion and the absence of the TcK1 gene were confirmed by PCR reactions. The obtained epimastigotes had no morphological and proliferation changes when maintained in rich medium and indicated the kinase absence is well-tolerated by replicating epimastigotes. As we observed eIF2α phosphorylation in starved TcK1-depleted parasites, it was clear that other kinases such as TcK2, or even TcK3 could phosphorylate eIF2α in these conditions.

Our results showed that cells lacking TcK1 still presented polysomes when submitted to nutritional stress, indicating that it has a direct effect on translation initiation. This appeared to occur independently of eIF2α phosphorylation as its levels were still detected in proliferating parasites. This contrasts with the effect of eIF2α T169A mutant observed previously, which had the same polysome levels and sustained the polysome levels upon incubation in TAU medium (Tonelli et al., 2011). However, a more evident decrease in eIF2α phosphorylation seen at 8 h after cell inoculation in fresh medium as shown in the quantification in Figure 4B, suggested that this effect occurs through eIF2α phosphorylation.

More relevant was that we noticed that *Tc*K1-depleted parasites showed reduced amounts of DHH1 granules upon full starvation. This result was confirmed by decreased levels of PABP1 found in granules. It is known that DHH1 prevents the binding of the ternary complex to the 5′end of the mRNA to form the 43S pre-initiation complex, inducing the decapping and mRNA degradation (Zeidan et al., 2018). DHH1 was also proposed to sense codon usage during translation elongation (Radhakrishnan et al., 2016), which is shown to perform a key control in *T. brucei* protein translation (Jeacock et al., 2018). PABP1 was found to interacts with several components of SG in *T. cruzi* and its differential accumulation could be a direct, or indirect action of TcK1 activity due to its phosphorylation state (de Melo Neto et al., 2018). Therefore, our findings suggested *Tc*K1 could have a role in controlling the gene expression in *T. cruzi*. In fact, the differences in the differentiation of epimastigotes to metacyclics, invasion capability of trypomastigotes and proliferation of amastigotes, could be attributed to differences in protein expression between control and mutated cells (Holetz et al., 2010).

The increased metacyclogenesis observed in the TcK1-KO compared to controls contrast with our previous results showing that the T169A mutant had reduced differentiation (Tonelli et al., 2011). These different phenotypes may be explained because eIF2α remains phosphorylated in TcK1-KO under nutritional stress when metacyclogenesis was induced. As metacyclogenesis was inhibited in the *Tc*K2 knockouts (da Silva Augusto et al., 2015), we can speculate that *Tc*K1 mutant has additional functions, which would promote increased differentiation.

Our results did not explain how TcK1 affected the DHH1 granule formation, but it could be related to the increased polysome levels. It is widely known that the formation of stress granules (SG) is associated with dramatic changes in gene expression coupled to differential translation, mRNA processing and mRNP granules formation (Guzikowski et al., 2019). Indeed, in some situations, it seems that there is a balance between the amount of mRNA engaged in translation with polysome formation, and the levels of SG formation. Furthermore, the composition of the different SG changes in different conditions (Guzikowski et al., 2019). SG and also P-bodies are rapidly formed in response to stress, as fast as the levels of mRNAs disengage from translation (Kershaw and Ashe, 2017). The composition of these granules varies but some components overlap in both granules, as is the case of DHH1 protein (Guzikowski et al., 2019). In agreement with our findings, the formation of SG may occur independently of eIF2α phosphorylation but through the action of GCN2. This protein kinase affects a myriad of cellular processes (Castilho et al., 2014). For example, situations that affect translation initiation via the eIF4F complex can lead to SG formation independently of eIF2α phosphorylation (Mahboubi and Stochaj, 2017). There is a crosstalk between the GCN2 pathway and other pathways involved in stress responses to nutrient starvation to avoid cell death, involving TOR (Cherkasova and Hinnebusch, 2003) and Snf1 (Cherkasova et al., 2010) kinases in yeast. In permanently activated GCN2, both P-bodies and SG were highly increased after nutritional stress, specifically under glucose deprivation. The granules became larger, brighter and more frequent in cells expressing constitutively active GCN2 compared to control during glucose deprivation in yeasts (Buchan et al., 2008). Aulas and colleagues vastly explored the effect of diverse stressful stimuli on SG formation in knockout cells of all 4 eIF2α kinases in HAP1 cells and its effect on eIF2α phosphorylation (Aulas et al., 2017). Surprisingly, the levels of phospho-eIF2α were not altered, except under UV-stress in GCN2 depleted cells, where it was abolished. Furthermore, GCN2 was shown to induce autophagy that clear the SG (Battu et al., 2018).

In conclusion, our findings demonstrate the role of the *T. cruzi* ortholog of GCN2, *T*cK1, in controlling several steps of *T. cruzi* life cycle through modulating the formation of SG. It does not perform its canonical function and does not affect the levels of phosphorylated eIF2α under nutritional stress. *Tc*K1 may have other potential substrates that have yet to be identified but may be related to the phenotypes observed. The elucidation of the mechanism by which *Tc*K1 modulates the formation of SG, as well as their role in the parasite, may help to understand the intricate regulatory network in this and other trypanosomes.

## Data Availability Statement

The raw data supporting the conclusions of this article will be made available by the authors, without undue reservation, to any qualified researcher.

## Ethics Statement

The animal study was reviewed and approved by the Comite de Ética em Pesquisa da Universidade Federal de São Paulo under protocol 266629/2015.

## Author Contributions

AM designed and characterized the TcK1-KO line, performed the polysome analyses, quantified the DHH1 RNP structures and wrote the paper. NM help designed the mutations, performed the metacyclogenesis and infections experiments and analysis, and discussed the results. GS generated and verified the *T. cruzi* lineage expressing Cas9 and helped in the characterization of the TcK1 mutation. MA performed DNA analysis experiments, growth curves, and maintained the parasite. FH helped with the polysome experiments and stress granule analyses. PB-C performed the Western blots using the α-phospho eIF2α antibodies and helped in the writing of the manuscript. SS obtained the funds, conducted the work strategy, analyzed the data, performed the phylogenetic analysis, and wrote the paper.

### Conflict of Interest

The authors declare that the research was conducted in the absence of any commercial or financial relationships that could be construed as a potential conflict of interest.
