# Supplementary material for: GCN2-Like Kinase Modulates Stress Granule Formation During Nutritional Stress in Trypanosoma cruzi
Source: Front Cell Infect Microbiol. 2020 Apr 16;10:149. doi: 10.3389/fcimb.2020.00149 (PMC7176912; doi:10.3389/fcimb.2020.00149)
Supplement: Supplementary file 1 [file Data_Sheet_1.PDF]

Supplementary Material

GCN2-like kinase phosphorylates *Trypanosoma cruzi* eIF2α during nutritional stress and modulates stress granule formation during parasite growth and differentiation

Amaranta Muniz Malvezzi[1]; Mirella Aricó Vieitas Costa[1]; Normanda Sousa Melo[1]; Gregory Pedroso dos Santos[1]; Paula Bittencourt-Cunha[1]; Fabiola Barbieri Holetz[2]; Sergio Schenkman[1]

1 Supplementary Tables

Table S1. Oligonucleotide sequences used in this work

**Primer**	**Sequence**
**Tck1-Primer sgRNA48**	5'-GAGGCCGGAGAATTGTAATACGACTCACTATAGGGAGAGTATAG CTGAGGGGCAGACAGGTTTTAGAGCTAGAAATAGCAAG 3'
**TcK1-Primer Forward for donor**	5'-CGGAAAAAAAAAAAAAAAAAAACGAGGAAGAGAGAAGGAAATAA AAGGAAGCAACAAGTCAAAGCATCGTATTAAGTTTTATGGCCAAG CCTTTGTCTCA 3'
**TcK1-Primer Reverse for donor**	5'-GAATCCCTCGAGCTTCACAACCTCAATGCCGTCATAGTTCTGCTTC AAAAACTCGATCTCATCCTCTATGACGTCACTGATTAGCCCTCCCAC ACATAAC
**Common Reverse Primer for sgRNA**	5'-AAAAAAGCACCGACTCGGTGCCACTT
**5K1fowApal**	5'-GGGCCCACGCCGTGTGACGGCAGAACG
**BSDFow**	5'-ATGGCCAAGCCTTTGTCTCAA
**BSDRev**	5'-TTAGCCCTCCCACACATAACCA
**CDSK1FowNdel**	5'-CATATGGTTTTTACTGAGGAATG
**1Tck1KDRevXhoI**	5'-CTCGAGCTCTCCGTCTCTTCTTCTGAAA
**sgRNAThre169v4**	5'-GGAGGCCGGAGAATTGTAATACGACTCACTATAGGGAGAGTTC CGTACACGGAAATTACGGTTTTAGAGCTAGAAATAGCAAG
**eIF2Donnorv2**	5'-GGCAATCATGAGGGTATTATTCCGTACACGGAAATTGCGCGCATC CGCATTCGGGCCATTGGAAAAGTTATCAAGGTAGG

Table S2 Sequences used for phylogenetic analysis

**Species**	**TriTrypDB.org Access number**
Blechomonas ayalai B08-376	Baya_024_0680
Crithidia fasciculata	CFAC1_130005500
Endotrypanum monterogeli	EMOLV88_110005300
Leishmania aethiopica	LAEL147_000146600
Leishmania amazonensis	LAMA_000170900
Leishmania arabica	LARLEM1108_110005500
Leishmania braziliensis MHOM/BR/75/M2903	LBRM2903_190007400
Leishmania braziliensis MHOM/BR/75/M2904	LbrM.19.0260
Leishmania donovani BPK282A1	LdBPK_110060.1
Leishmania donovani CL-SL	LdCL_110005500
Leishmania donovani strain LV9	LdBPK.11.2.000060:pseudogene
Leishmania enriettii strain *LEM3045*	LENLEM3045_110006000
Leishmania gerbilli strain LEM452	LGELEM452_110005500
Leishmania infantum JPCM5	LINF_110005500
Leishmania major strain Friedlin	LmjF.11.0060
Leishmania major strain LV39c5	LMJLV39_110005500
Leishmania major strain SD 75.1	LMJSD75_110005500
Leishmania mexicana MHOM/GT/2001/U1103	LmxM.11.0060
Leishmania panamensis MHOM/COL/81/L13	LPAL13_190007500
Leishmania panamensis strain MHOM/PA/94/PSC-1	LPMP_110060
Leishmania sp. MAR LEM2494	LMARLEM2494_110005500
Leishmania tarentolae Parrot-TarII	LtaP11.0060
Leishmania tropica L590	LTRL590_110005600
Leishmania turanica strain LEM423	LTULEM423_110005500
Leptomonas pyrrhocoris H10	LpyrH10_18_0070
Leptomonas seymouri ATCC 30220	Lsey_0397_0010
Paratrypanosoma confusum CUL13	PCON_0033780
Trypanosoma brucei brucei TREU927	Tb11.02.5050b
Trypanosoma brucei brucei TREU927	Tb927.11.7210
Trypanosoma brucei gambiense DAL972	Tbg972.11.8150
Trypanosoma brucei Lister strain 427	Tb427tmp.02.5050
Trypanosoma brucei Lister strain 427 2018	Tb427_110077000
Trypanosoma congolense IL3000	TcIL3000_0_47340
Trypanosoma cruzi Dm28c 2014	TCDM_00579
Trypanosoma cruzi Dm28c 2014	TCDM_00580
Trypanosoma cruzi DM28c 2017A	BCY84_04808
Trypanosoma cruzi DM28c 2017B	BCY84_12930
Trypanosoma cruzi DM28c 2018	C4B63_70g58
Trypanosoma cruzi marinkellei strain B7	Tc_MARK_567
Trypanosoma cruzi strain Y	NMZO01000706.1:2,035..5,676
Trypanosoma cruzi Sylvio X10/1-2012	TCSYLVIO_001721
Trypanosoma cruzi TCCA	C3747_43g42
Trypanosoma cruzi TCCB	C3747_44g8
Trypanosoma evansi strain STIB 805	TevSTIB805.11_01.7410
Trypanosoma gray	DQ04_01851080
Trypanosoma theileri isolate Edinburgh	TM35_000162570

2 Supplementary Figures

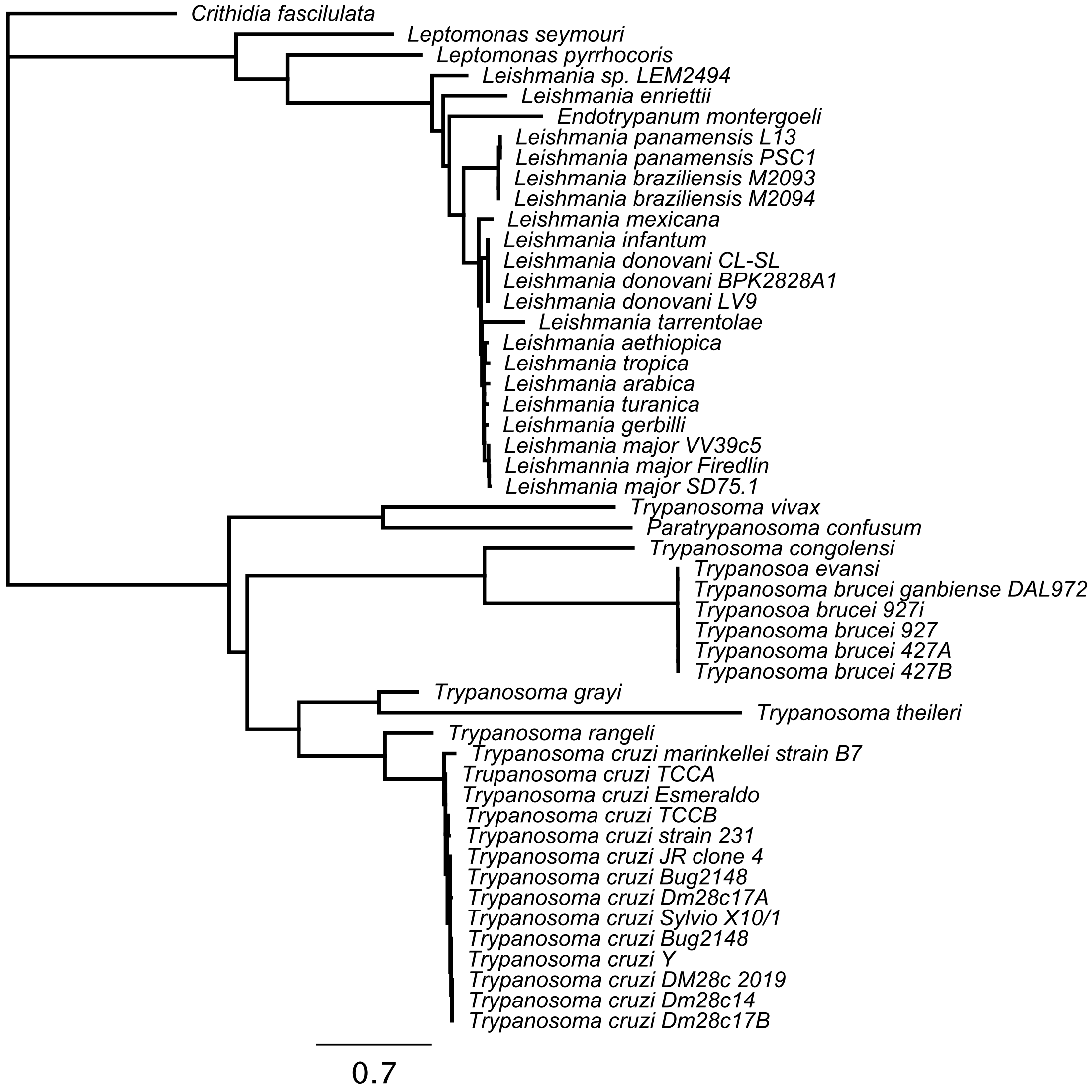

Supplementary Figure 1. Phylogenetic tree of alignment of the insert regions of the KD domain of Kinetoplastidae GCN2 homologues. The nucleotide sequences corresponding to the inserts of KDs were aligned with MUSCLE (10 iterations) and the phylogenetic tree constructed using PHYML with K80 nucleotide substitution model. The access numbers for each species can be found in Supplementary Table 2.

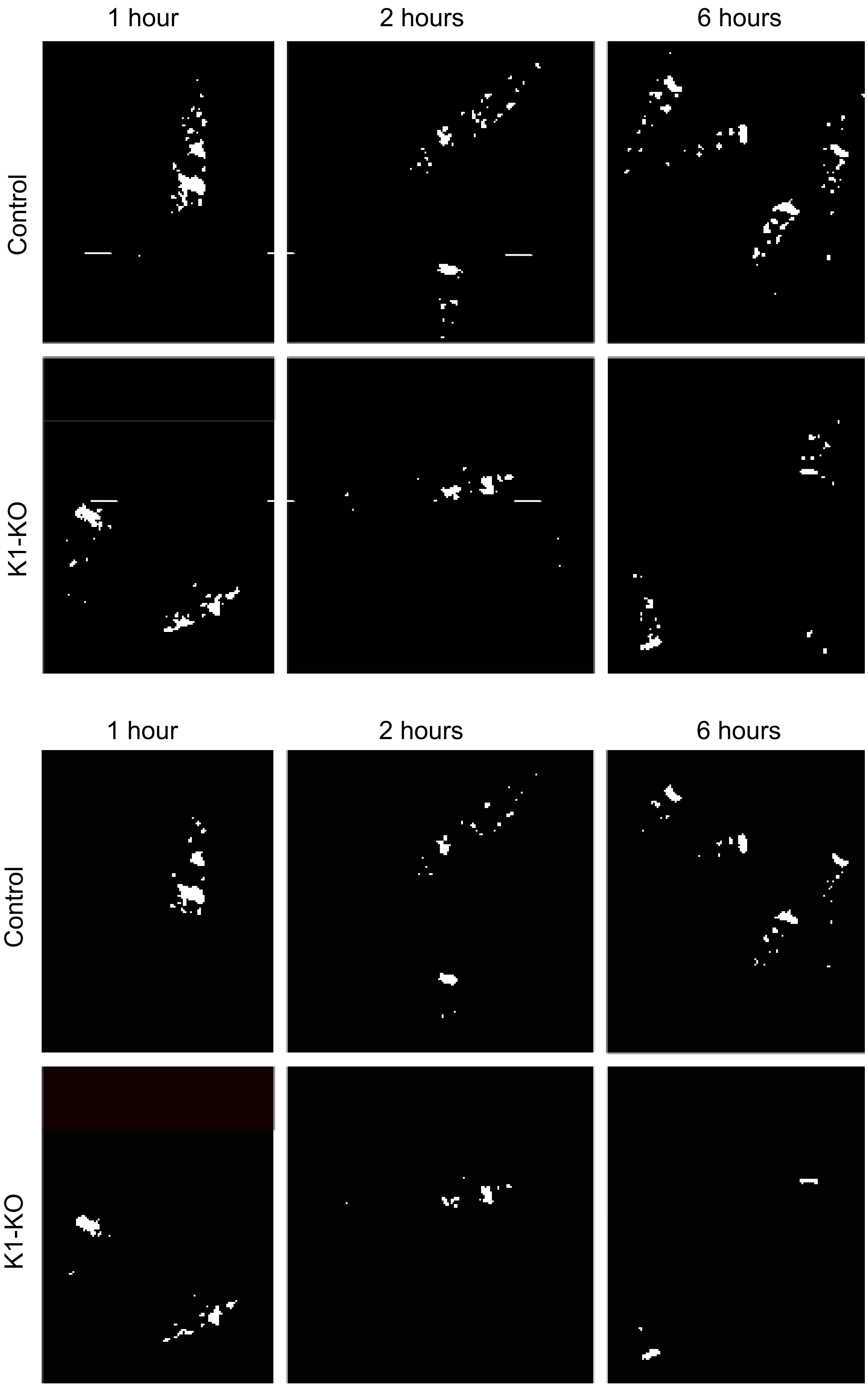

Supplementary Figure 2. Threshold analysis of the stress granules of control and TcK1 depleted parasites. The images were taken from figure 5B and submitted to two different levels of threshold using Affinity Designer software.
